# Novel Mixed-Type Inhibitors of Protein Tyrosine Phosphatase 1B. Kinetic and Computational Studies

**DOI:** 10.3390/molecules22122262

**Published:** 2017-12-20

**Authors:** Marie Jazmín Sarabia-Sánchez, Pedro Josué Trejo-Soto, José Miguel Velázquez-López, Carlos Carvente-García, Rafael Castillo, Alicia Hernández-Campos, Claudia Avitia-Domínguez, Daniel Enríquez-Mendiola, Erick Sierra-Campos, Mónica Valdez-Solana, José Manuel Salas-Pacheco, Alfredo Téllez-Valencia

**Affiliations:** 1 Facultad de Medicina y Nutrición, Universidad Juárez del Estado de Durango, Av. Universidad y Fanny Anitúa S/N, Durango, Durango C.P. 34000, Mexico; marie_sarabia@hotmail.com (M.J.S.-S.); avitiaclaudia@gmail.com (C.A.-D.); qfb.dan.enriquez@hotmail.com (D.E.-M.); 2 Facultad de Química, Departamento de Farmacia, Universidad Nacional Autónoma de México, Ciudad de México C.P. 04510, Mexico; Piter_jo@comunidad.unam.mx (P.J.T.-S.); miguelzx42@hotmail.com (J.M.V.-L.); krbnt@hotmail.com (C.C.-G.); rafaelc@unam.mx (R.C.); hercam@unam.mx (A.H.-C.); 3 Facultad de Ciencias Químicas, Universidad Juárez del Estado de Durango, Av. Artículo 123 S/N Fracc. Filadelfia, Gómez Palacio, Durango C.P. 35010, Mexico; ericksier@gmail.com (E.S.-C.); valdezandyval@gmail.com (M.V.-S.); 4 Instituto de Investigación Científica, Universidad Juárez del Estado de Durango, Av. Universidad S/N, Durango, Durango C.P. 34000, Mexico; jsalas_pacheco@hotmail.com

**Keywords:** protein tyrosine phosphatase 1B, type 2 diabetes, benzimidazole derivatives, enzyme inhibition, docking, molecular dynamics

## Abstract

The Atlas of Diabetes reports 415 million diabetics in the world, a number that has surpassed in half the expected time the twenty year projection. Type 2 diabetes is the most frequent form of the disease; it is characterized by a defect in the secretion of insulin and a resistance in its target organs. In the search for new antidiabetic drugs, one of the principal strategies consists in promoting the action of insulin. In this sense, attention has been centered in the protein tyrosine phosphatase 1B (PTP1B), a protein whose overexpression or increase of its activity has been related in many studies with insulin resistance. In the present work, a chemical library of 250 compounds was evaluated to determine their inhibition capability on the protein PTP1B. Ten molecules inhibited over the 50% of the activity of the PTP1B, the three most potent molecules were selected for its characterization, reporting Ki values of 5.2, 4.2 and 41.3 µM, for compounds **1**, **2**, and **3**, respectively. Docking and molecular dynamics studies revealed that the three inhibitors made interactions with residues at the secondary binding site to phosphate, exclusive for PTP1B. The data reported here support these compounds as hits for the design more potent and selective inhibitors against PTP1B in the search of new antidiabetic treatment.

## 1. Introduction

In its sixth edition, the Atlas of Diabetes reported 415 million diabetics worldwide, surpassing in half the time the twenty year projection [1,2]. Type 2 diabetes is the most frequent type of the disease; it is characterized by a defect in the secretion of insulin and resistance in its target organs. For its treatment there are several hypoglycemic agents, these have different mechanisms of action such as an increase in insulin production, decrease of the hepatic glucose production, limiting the absorption of postprandial glucose, and inhibiting gluconeogenesis [3]. Nevertheless, after 3 or 4 years of treatment the efficacy of these drugs is diminished, even with combinations among them, and insulin administration becomes necessary [4]. Therefore, there is an urgency for new drugs with other mechanisms of action that can provide different alternative treatments for type 2 diabetes.

To this end, one of the main strategies consists in promoting the action of insulin [5], and the attention has been focused in the protein tyrosine phosphatase 1B (PTP1B), a protein which overexpression and increase in its activity has been related in many studies with insulin resistance [6,7,8]. PTP1B works specifically by dephosphorylating residues of phosphotyrosine both from the insulin receptor (IR) and insulin receptor substrate (IRS) [9]. In a recent study, Munc18c was discovered as a substrate for the PTP1B, it is related to the regulation of glucose transporter GLUT4 in adipocytes, allowing or impeding, the insertion of the vesicle into the membrane [10]. These and other evidences [11,12,13] validate this enzyme as a potential therapeutic target against type 2 diabetes.

Since the establishment of the PTP1B as a biological target, there has been an effort to obtain inhibitors of its activity [14,15]. Different strategies such as diverse computational techniques, natural products research, and medicinal chemistry have been applied in the search for PTP1B inhibitors [16,17,18,19,20]. The first efforts to obtain inhibitors consisted in the search for phosphotyrosine (pTyr) mimetics such as difluoromethylene phosphate (DFMP) [21,22,23,24], carboxylic acids [25,26,27,28], 1,2,5-thiazolidin-3-one 1,1-dioxide (TZD), and the (*S*)-isomer of isothiazolidinone ((*S*)-IZD) [24,29], which achieve different degrees of inhibition of the enzyme. Furthermore, natural products and some derivatives have been reported too [30,31], as well as small molecules optimized from the previous ones [23,32]. Nevertheless, finding a potent, selective and with good oral availability molecule is still a challenge to be overcome.

The highly conserved catalytic site in the phosphatases family hinders the finding of a selective molecule, especially against its closest homologous T-cell protein tyrosine phosphatase (TCPTP) [33,34]. One strategy is to seek interactions with specific sites of the PTP1B [35,36,37]. In this sense, the enzyme has two aryl phosphate binding sites, a catalytic site with high affinity that contains the Cys215, and another one with low affinity that contains the Arg24 and Arg254. The former, denominated as site B, is specific for PTP1B. A few molecules based on this concept have reached clinical trials, among which ertiprotafib, ISIS 113715 and trodusquemine may be highlighted [38,39,40], however they did not continue to later stages.

In the present work, a chemical library composed by 250 compounds was evaluated to determine their inhibition capabilities on the PTP1B. The three most potent molecules were selected for further characterization, including their mechanism and inhibition constants. Structural studies of the PTP1B-inhibitor interaction were performed through docking and molecular dynamics simulations as well as an estimation of their drug-like and toxicological properties.

## 2. Results and Discussion

### 2.1. Compounds Screening

**Figure 1:**
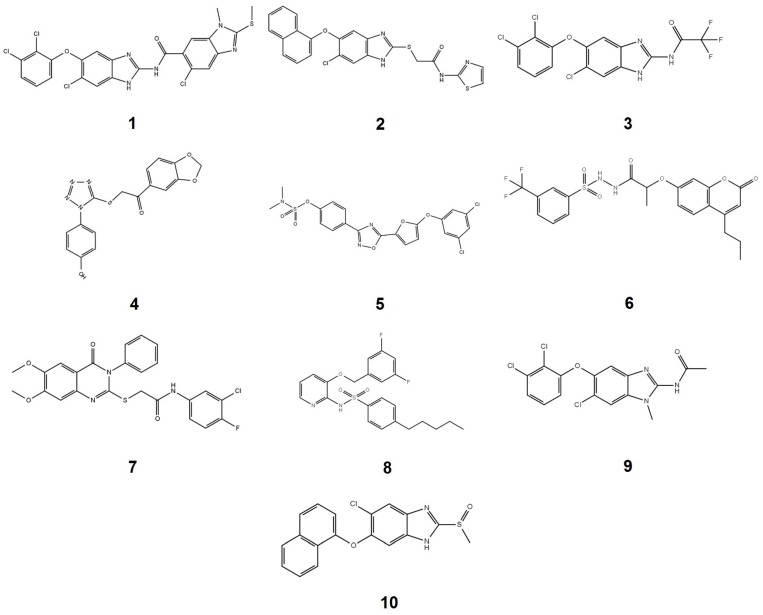
Chemical structures of the ten most potent PTP1B inhibitors. The number of each compound corresponds to that indicated in Table 1.

With the aim of finding new compounds capable of inhibiting PTP1B activity, a chemical library of 250 small molecules was evaluated at 200 µM. From the total of compounds evaluated, 26 inhibited the enzyme activity by more than 60%, 26 of them between 31–59% (Table S1), and the rest below 30%. The characteristics of the ten most potent compounds are shown in Table 1, and their structures in Figure 1.

According to their structure, the three most potent PTP1B inhibitors (compounds **1**, **2** and **3**) all contain a benzimidazole nucleus. The analysis suggests that bulky substituents are required at positions 2 (**1** vs. **9** and **10**) and 5 (**1** vs. **10**) of the benzimidazole skeleton to increase their inhibition capability. The presence of the benzimidazole nucleus in the structure of the PTP1B inhibitors has been reported before, nevertheless, due to their substituents, they showed permeability and specificity problems with respect to other phosphatases [41,42,43].

Regarding molecules **5**, **6** and **8** they have in their structure substituents that have been reported before in PTP1B inhibitors [28]. Molecules **4** and **7** represent a new chemical nucleus, which extends the structural diversity of molecules that inhibit PTP1B reported to date. Finally, molecules **1**, **2** and **3** were selected to characterize their inhibition mechanism by enzymatic kinetics, docking and molecular dynamics.

### 2.2. Kinetic Studies

**Figure 2:**
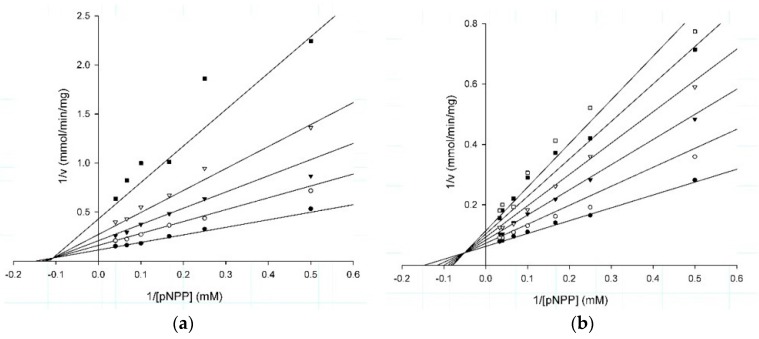

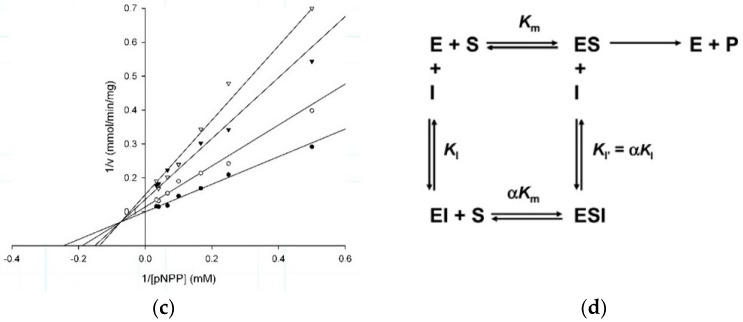
Lineweaver-Burk plots of (**a**) compound **1** at 0 (filled circles), 3 (open circle), 6 (filled triangle), 10 (open triangle), and 20 µM (filled squares); (**b**) compound **2** at 0 (filled circles), 2 (open circle), 4 (filled triangles), 6 (open triangles), 8 (filled squares), and 10 µM (open squares); and (**c**) compound **3** at 0 (filled circles), 20 (open circle), 50 (filled triangles), and 70 µM (open triangles); (**d**) kinetic model for mixed type inhibition. In the scheme, E corresponds to free enzyme; S is the substrate; ES is the enzyme-substrate complex; EI corresponds to the enzyme-inhibitor complex; ESI is the enzyme-substrate-inhibitor ternary complex; and P is the reaction product.

Analysis of the plots at different substrate and fixed inhibitor concentrations indicated that the three compounds showed a mixed type inhibition mechanism (Figure 2a–c). This suggests that the three molecules are able to recognize both the free enzyme and the enzyme-substrate complex, generating the enzyme-substrate-inhibitor ternary complex, which is inactive [46] (Figure 2d). Something interesting to highlight is that compound **1** had an α value close to 1 suggesting that it recognizes, almost with the same affinity, both the free enzyme and the enzyme-substrate complex [46], whilst in compounds **2** and **3** this value was close to 3, suggesting a three times lower affinity with respect to the enzyme-substrate complex. These results suggest that the noncompetitive component of these inhibitors is stronger than the competitive component into inhibition mechanism, where the values of Ki and IC_50_ in our study were very similar [47]. The kinetic parameters were obtained from the adjustment of the data to the correspondent equation (Table 2).

The ChEMBL database from the Bioinformatics European Institute [48], reported 5854 compounds active against PTP1B. Of interest of this work, 30 of them show a reported mixed type inhibition mode, but only eight presented a Ki value lower than 5 µM [49,50,51,52,53,54,55,56,57,58,59,60,61]. Something important is that the chemical structures of these inhibitors are totally different from those reported here, and include DMFS derivatives [58], benzoic acid-based derivatives [57], insulin-mimetic selaginellins [59], pentacyclic acid triterpenoids [52], and oleanilic acid derivatives [61]. Also interesting is that α value reported was in the same range as for compounds **1**, **2**, and **3**.

With respect to available drugs for the treatment of type 2 diabetes, classified in a general way as sulfonylureas, meglitidines, biguanides, thiazolidinediones, α-glucosidase inhibitors, glucagon-like peptide-1 receptor agonists, dipeptidyl peptidase-4 inhibitors, sodium glucose transporter-2 inhibitors, synthetic amylin analogues, and dopamine-2 agonists [62,63], none of them are benzimidazole derivatives, on the contrary, they are made up of different chemical groups such as sulfonylureas, guanidines, thiazolidinediones, disaccharides, glucose derivatives, peptidomimetics, among others [63]. The above makes the compounds **1**, **2** and **3** novel structural proposals.

After kinetic studies, the binding mode and the type of interactions between the inhibitors and the PTP1B were analyzed by docking and molecular dynamics studies.

### 2.3. Molecular Docking

Before the molecular docking of the inhibitors, the protocol was validated through the binding mode of the inhibitor reported in the crystallographic structure used [64]. The RMSD value obtained from the modeling using Glide and the crystallographic complex was of 0.24 Å, which indicated that the docking protocol was done correctly (data not shown). After this, the three inhibitors were docked, obtaining binding energies of −4.2, −5.0 and −4.5 Kcal/mol for compounds **1**, **2**, and **3**, respectively.

As for their binding modes, the three compounds formed interactions with residues from the secondary binding site to phosphate (Arg24, Arg254, Gly259, Gln262 and Asp48) [65]. In the three cases, the molecules block the cavity of the catalytic site without interacting with the signature residues of phosphatases.

**Figure 3:**
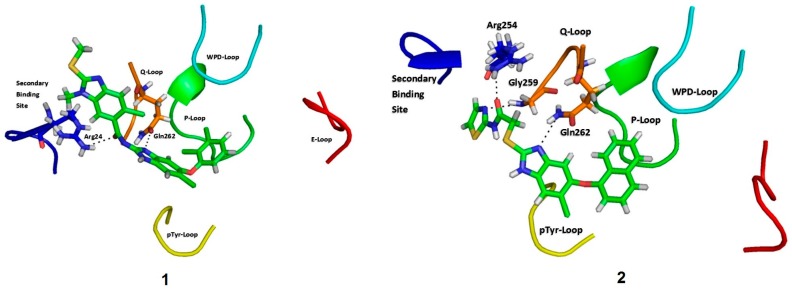

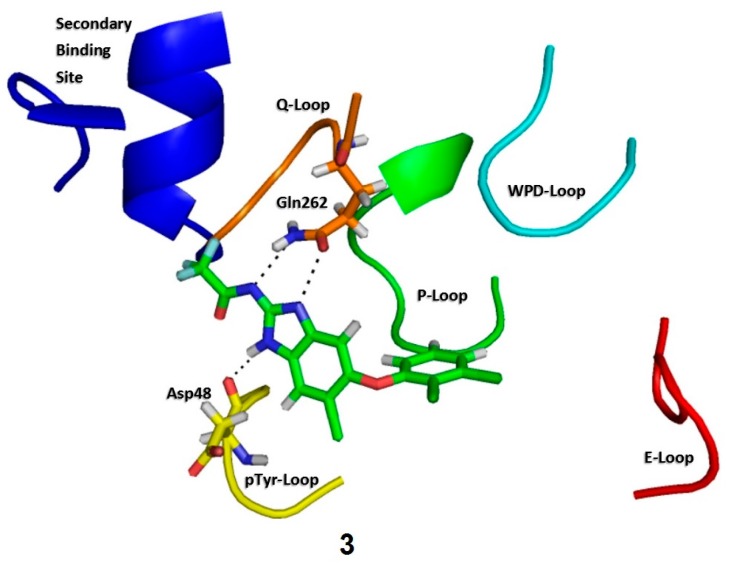
Binding mode of compounds **1**, **2** and **3** in PTP1B. Loops are highlighted as follows: P loop (green), WPD loop (cyan), Q262 loop (orange), pTyr46 loop (yellow), and E loop (red). Secondary phosphate binding site is highlighted in blue.

The interaction with Asp48 has been reported before in different crystallographic structures of PTP1B in complex with other benzimidazole derivatives [41,42,43]. Additionally the interaction of the benzimidazole nucleus with the Gln262 found in the three inhibitors has been also reported [66,67], (Figure 3). A more detailed analysis of the interactions between these molecules and the enzyme were performed by molecular dynamic studies, the results are described below.

### 2.4. Molecular Dynamics Simulations

The complexes obtained by molecular docking were submitted to a simulation of 10 ns. The total energy variation plots showed that the energy variation was around −12 Kcal/mol, which indicate that the average energy remains constant and there is structural stability of the complexes (Figure S1). The RMSD analysis showed that the three complexes had fluctuations in the first 3500 ps, achieving the stability from the 4000 ps up to the 10,000 ps without exceeding a 0.30 Å variation (Figure S2). It also was observed the influence of the inhibitors over the protein, with the variation of the RMSD in comparison with the protein alone.

The analysis of the binding energies showed that the compound **1** presented the best global binding energy, followed by compounds **2** and **3**, which is in accordance with the inhibitory activity observed in the kinetic studies. The same situation was repeated in the individual values of the different components of the global energy, except in the electrostatic one, where compound **2** obtained the highest value (Table 3).

**Figure 4:**
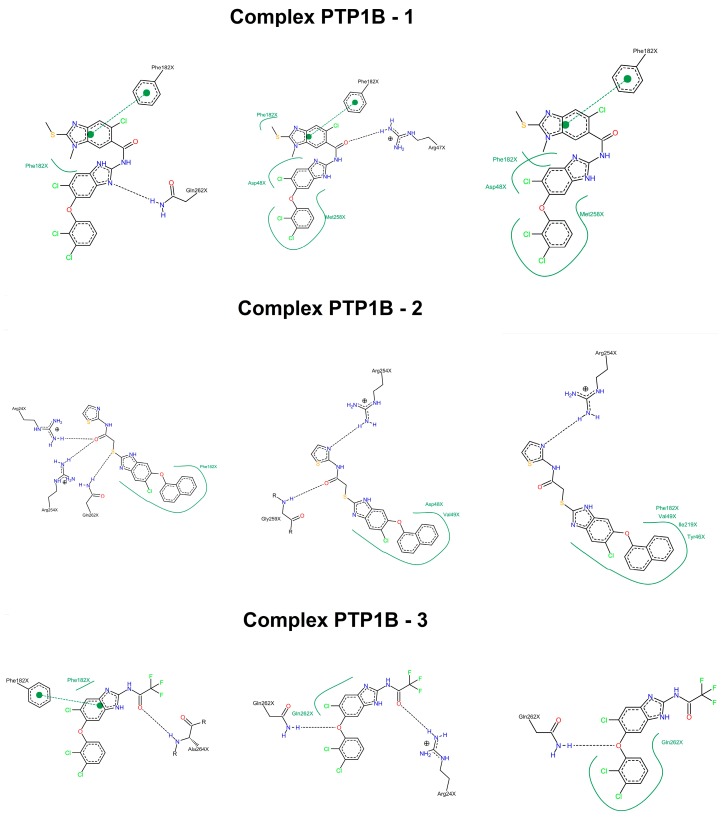
Two dimensional maps of interaction from the complexes PTP1B-**1**, PTP1B-**2,** and PTP1B-**3**. The image shows interactions at different times during entire dynamics: beginning, stabilized (4 ns), and final (10 ns). Hydrogen bonds between protein and ligand are drawn as dashed lines. Hydrophobic contacts are represented by means of spline sections highlighting the hydrophobic parts of the ligand and the name of the contacting amino acid. Maps were generated in Server Poseview (http://proteinsplus.zbh.uni-hamburg.de/#poseview).

The structural analysis along the simulation time showed that the three molecules formed interactions, being the most important, the hydrophobic type interaction with the Asp48 and compound **1** (70% of occupancy), meanwhile compound **2** interacted with Phe182 (87% of occupancy). Compound **3** formed hydrogen bonds with Ala264, Gln262, and Arg24, as well as a hydrophobic interaction with Phe182, all of them with occupancy of 40% (Figure 4). Something interesting to highlight is that the interactions formed by the three inhibitors include important residues for the enzyme function like Gln262 and the Asp48, without having interactions with the denominated signature residues of the phosphatases (H/V)CXXGXXR(S/T) [34,68]. In this context, several studies have shown that selectivity against TCPTP can be achieved by interactions with residues such as Arg24, Arg47, Asp48, Arg254, Met258, and Gln262 [35,65]. Additionally, we investigated their binding mode in TCTPT by molecular docking, finding that the three compounds made interactions with different residues of the enzyme (Figure S3). In conclusion, computational studies suggest that these inhibitors could be selective for PTP1B.

### 2.5. Physicochemical and Drug-Like properties

**Figure 5:**
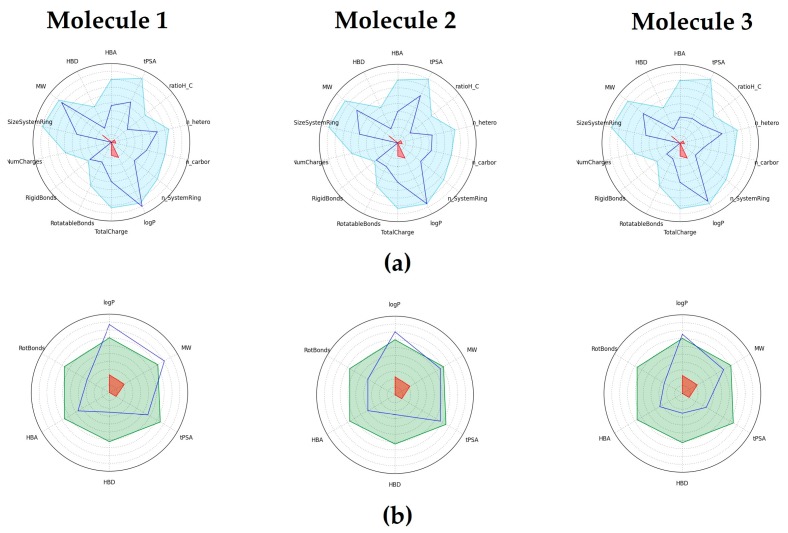
(**a**) Scheme of PhysChem Filter Positioning, compound values (blue line) should fall within the drug-like filter area (light blue); (**b**) Scheme of Oral Absorption Estimation, compound values (blue line) should fall within RO5 and Veber rules area (light green). The logarithm of the partition coefficient between n-octanol and water, logP; Molecular Weight, MW; Hydrogen Bond Donnors, HBD; Hydrogen Bond Acceptors, HBA; topological Polar Surface Area, tPSA; het/carbon atoms ratio, ratioH/C; Number of Heteroatoms, n_hetero; Number of Carbon Atoms, n_carbor; Number of Smallest Set of Smallest Rings, n_SystemRing.

The in silico evaluation of the physicochemical and drug-like parameters suggested that these molecules possess the necessary chemical features to potentially have an acceptable oral absorption [69,70,71] (Table 1 and Figure 5).

### 2.6. Toxicological Evaluation

An important point to analyze during the development of any new drug is the toxicological profile of the molecule. In this matter, using different softwares available online, a detailed study to predict the toxicological potential of these three inhibitors was performed. The estimation of the lethal dose 50 (LD_50_) suggested that the three molecules are moderately toxic. Nevertheless, according to software evaluation, there are no toxic fragments reported in their structure, neither biological targets that can denote toxicity. With respect to their mutagenic, tumorigenic, irritability and reproductive effects, the only molecule that did not show any of these features was compound **2** (Table 4).

Taking into account all the data presented, compound **2** is the most viable option to continue with its optimization since it showed the best kinetic and predicted physicochemical and toxicological features. However, compounds **1** and **3** are still interesting structures that provide important information for the design of new inhibitors.

## 3. Materials and Methods

### 3.1. General Information

The reagents used were purchased from Sigma-Aldrich (St. Luis, CA USA), kinetic analysis were performed in a diode array spectrophotometer model 8453 from Agilent (Santa Clara, CA, USA).

### 3.2. Compounds

The tested chemical library was composed of an in-house set of 100 compounds and 150 small molecules of the Fragment Library and HitFinder^TM^ collection from Maybridge (Waltham, MA, USA). Compounds **4** to **8** belong to Maybridge with the identification codes HTS 01664 for ′1-(1,3-benzodioxol-5-yl)-2-{[1-(4-hydroxyphenyl)-1*H*-1,2,3,4-tetraazol-5-yl]sulfanyl}-1-ethanone (**4**); SP 00892 for 4-{5-[5-(3,5-dichlorophenoxy)-2-furyl]-1,2,4-oxadiazol-3-yl}phenyl-*N*,*N*-dimethylsulfamate (**5**); RJF 01991 for ′*N*′1-{2-[(2-oxo-4-propyl-2*H*-chromen-7-yl)oxy]propanoyl}-3-(trifluoromethyl)benzene-1-sulfonohydrazide (**6**); HTS 02534 for ′*N*-(3-chloro-4-fluorophenyl)-2-[(6,7-dimethoxy-4-oxo-3-phenyl-3,4-dihydro-2-quinazolinyl)sulfanyl]acetamide (**7**); RH 02067 for ′*N*-{3-[(3,5-difluorobenzyl)oxy]pyridin-2-yl}-4-pentylbenzenesulfonamide (**8**). The general synthesis method for compounds **1**, **2**, **3**, **9** and **10** is outlined below.

Compounds **1**–**3** and **9** were prepared from the appropriate benzimidazole-2-amine and the adequate acid or anhydride under the guidelines of our synthetic procedure previously reported for similar benzimidazole derivatives [74,75]. Briefly, for compound **1**: the substituted benzimidazol-2-amine was reacted with 5-chloro-1-methyl-2-(methylthio)-6-carboxylic acid, previously treated with 1,1′-carbonyldiimidazole in DMF at room temperature for 2 h; then, the reaction mixture was heated at 140 °C under MW irradiation for 30 min. For compounds **3** and **9**: the substituted benzimidazol-2-amine was reacted with trifluoroacetic anhydride (compound **3**) or acetic anhydride (compound **9**) at 0 °C to r.t. in CH_2_Cl_2_ or CHCl_3_; compound **2** was prepared from 6-chloro-5-(1-naphtyloxy)-1*H*-benzimidazole-2-thiol [76] and 2-chloro-*N*-(thiazol-2-yl)acetamide in acetone at 0 °C [77]. Compound **10** was obtained as previously reported [78].

*5-Chloro-N-[6-chloro-5-(2,3-dichlorophenoxy)-1H-benzimidazol-2-yl]-1-methyl-2-(methylthio)-1H-benz-imidazole-6-carboxamide* (**1**). Recrystallized from DMF/MeOH white solid (89%); m.p. 269-270 °C. ^1^H-NMR (DMSO-*d*_6_; 400 MHz): δ 2.75 (s, 3H, S-CH_3_); 3.72 (s, 3H, N-CH_3_); 6.69 (dd, 1H, *J* = 8.2 Hz, 1.2 Hz, H-6 dichlorophenoxy); 7.27 (t, 1H, *J* = 8.2 Hz, H-5 dichlorophenoxy); 7.33 (s, 1H, H-4′); 7.37 (dd, 1H, *J* = 8.4 Hz, 1.2 Hz, H-4 dichlorophenoxy); 7.68 (s, 1H, H-7′); 7.71 (s, 1H, H-5); 7.91 (s, 1H, H-7); 12.40 (bs, 1H, NH, int. D_2_O). ^13^C-NMR (DMSO-*d*_6_; 100 MHz): δ 14.48 (S-*C*H_3_); 30.56 (N-*C*H_3_); 110.93 (C-7 benzimidazole); 115.64 (C-6 dichlorophenoxy); 118.45 (C-4 benzimidazole); 118.59 (C-7′a benz-imidazole); 121.36 (C-2 dichlorophenoxy); 123.73 (C-5 benzimidazole); 124.69 (C-4 dichlorophenoxy); 127.79 C-6 benzimidazole); 129.11 (C-5 dichlorophenoxy); 133.23 (C-3 dichlorophenoxy); 135.63 (C-7a benzimidazole); 144.89 (C-3a benzimidazole); 145.04 (C-6′ benzimidazole); 148.58 (C-2′ benzimidazole); 155.10 (C-1 dichlorophenoxy); 157.27 (C-2 benzimidazole); 166.85 (*C*=O amide). EI-MS: *m*/*z* 565 (M^+^); HRMS (FAB^+^): 565.9752 [M + H]^+^ (Calcd for C_23_H_15_O_2_N_5_Cl_4_SH^+^ 565.9773).

*2-[6-Chloro-5-(1-naphthalyloxy)-1H-benzimidazol-2-yl]thio-N-(thiazol-2-yl)acetamide* (**2**). Recrystallized from methanol to give a beige solid (20% yield); m.p. 155-157 °C. ^1^H-NMR (400 MHz, DMSO): δ 4.37 (s, 2H, -CH_2_-). 6.64 (d, *J* = 7.6 Hz, 1H, H-2 naphtyloxy), 7.23 (d, *J* = 3.6 Hz, 1H, H-5 thiazolyl), 7.27 (s, 1H, H-7), 7.37 (t, *J* = 8.0 Hz, 1H, H-3 naphtyloxy), 7.48 (d, *J* = 3.6 Hz, 1H, H-4 thiazolyl), 7.61–7.57 (m, 2H, H-6 y H-7 naphtyloxy), 7.64 (d, *J* = 8.0 Hz, 1H, H-4 naphtyloxy), 7.72 (s, 1H, H-4), 7.99–7.95 (m, 1H, H-5 naphtyloxy), 8.27–8.22 (m, 1H, H-8 naphtyloxy), δ 12.70 (s, 1H, CONH). ^13^C-NMR (DMSO-*d*_6_; 100 MHz): δ 34.79 (SCH_2_), 109.88 (C-2 naphtyloxy), 113.78 (C-3 naphtyloxy), 119.12 (C-5 thiazolyl), 121.36 (C-8 naphtyloxy), 122.45 (C-4 naphtyloxy), 124.86 (C-5 or C-6), 126.02 (C-6 or C-7 naphtyloxy), 126.13 (C-6 or C-7 naphtyloxy), 126.85 (C-4a or C-8a naphtyloxy), 127.77 (C-5 naphtyloxy), 134.45 (C-4a or C-8a naphtyloxy), 137.76 (C-4 thiazolyl), 146.06 (C-3a or C-7a), 151.96 (C-3a or C-7a), 153.37 (C-2), 157.81 (C-2 thiazolyl), 166.22 (CONH). MS (DART): *m*/*z* (%): 467 ([M + H]^+^_,_ 15). HRMS (DART): Calcd for C_22_H_15_ClN_4_O_2_S_2_ [M + H]^+^: 467.04032, found: 467.04177.

*N-[6-Chloro-5-(2,3-dichlorophenoxy)-1H-benzimidazol-2-yl]-2,2,2-trifluoroacetamide* (**3**). Purified by washing with cold water. Beige solid; m.p. > 200 °C (d). ^1^H-NMR (400 MHz, DMSO) δ: 6.84 (dd, 1H, *J*_1_ = 8.3, *J*_2_ = 1.3 Hz, H-6′); 7.21 (s, 1H, H-4); 7.32 (t, 1H, *J* = 8.2 Hz, H-5′); 7.44 (dd, 1H, *J*_1_ = 8.1 Hz, *J*_2_ = 1.3 Hz, H-4′); 7.66 (s, 1H, H-7); 13.07 (bs, 1H, CO-N*H*). ^13^C-NMR (DMSO-*d*_6_; 100 MHz): δ 105.22 (C-4), 114.10 (C-7), 116.97 (C-6′), 117.52 (q, *J*_F-C_ = 287 Hz, -*C*F_3_), 120.37 (C-6), 122.15 (C-2′), 125.65 (C-4′), 127.03, 129.22, 129.36 (C-5′), 133.41 (C-3′), 147.07 (C-5), 153.98 (C-2), 154.17 (C-1’), 162.99 (q, *J*_F-C_ = 35 Hz, *C*O).

*N-[6-Chloro-5-(2,3-dichlorophenoxy)-1-methyl-1H-benzimidazol-2-yl]acetamide* (**9**). Recrystallized from ethanol, white crystals (84% yield); m.p. 237.5–238.9 °C. ^1^H-NMR (400 MHz, DMSO) δ: 2.16 (s, 3H, CO-CH_3_); 3.64 (s, 1H, N-CH_3_); 6.65 (d, 1H, *J*_1_ = 8.2 Hz, *J*_2_ = 0.9 Hz, H-6′); 7.24 (t, 1H, *J* = 8.2 Hz, H-5′); 7.36 (dd, 1H, *J*_1_ = 8.1 Hz, *J*_2_ = 1.3 Hz, H-4′); 7.42 (s, 1H, H-4); 7.88 (s, 1H, H-7); 10.97 (bs, 1H, CO-NH). ^13^C-NMR (DMSO-*d*_6_; 100 MHz): δ 23.43 (CO-*C*H_3_), 30.97 (N-*C*H_3_), 111.95 (C-4), 112.27 (C-7), 115.51 (C-6′), 119.52 (C-6), 121.28 (C-2′), 124.70 (C-4′), 129.16 (C-5′), 133.25, 140.41 (C-3′), 145.39 (C-4), 148.37 (C-2), 155.14 (C-1′), 170.57 (*C*O). EA. Calc.: C_16_H_12_Cl_3_N_3_O_2_: C, 49.96; H, 3.14; N, 10.9. Found: C, 49.82; H, 2.57; N, 10.6. HMRS (ESI) Calcd for C_16_H_12_Cl_3_N_3_O_2_ [M + Na]: 406.0073; found 406.

### 3.3. Expression and Purification of PTP1B

The region of the gene PTPN1 that encodes for PTP1B (residues 1–321) was synthetized by Integrated DNA Technologies and inserted in the pIDTSmart plasmid. Then, the gene was liberated by restriction reactions using Ndel and BamH1 enzymes and inserted into the overexpression vector pET28A. Afterwards, *E. coli* BLR strains were transformed for the overexpression of the protein. With this purpose, 500 mL of LB liquid culture medium was grown supplemented with Kanamycin (50 µg/mL) at 37 °C, once it reached an optical density of 0.9 at 600 nm, 1mM of IPTG was added to induce the overexpression, incubating four more hours. Right away, cells were cultured by centrifugation and lysed by sonication. The supernatant was passed through a Ni-agarose column and the enzyme was purified by an imidazole gradient. The fractions were analyzed by SDS-PAGE electrophoresis and those with the presence of the protein were pooled and concentrated with a Plus-70 centricon, immediately the enzyme was precipitated with ammonium sulfate (80% saturation).

### 3.4. Enzymatic Activity

The PTP1B activity was measured based on the Goldstein method [79]. The assay was performed with a final reaction volume of 500 µL in HEPES buffer (50 mM HEPES, 1mM DTT, 2 mM EDTA and 150 mM NaCl, pH 7.0), DMSO (10%) and p-nitrophenol phosphate (pNPP) as substrate (50 mM), the reaction was started with the PTP1B (1.5 µg/mL). After 30 min of incubation at 37 °C, the reaction was stopped by the addition of 500 µL of NaOH 5N reading the absorbance at 405 nm. The number of hydrolyzed moles of pNPP was determined using the molar extinction coefficient of the product pNP (18,500 M^−1^ cm^−1^).

### 3.5. Inhibition Assays

Inhibition assays were performed under the above described conditions, adding to the reaction each one of the molecules at a final concentration of 200 µM. The concentration that inhibits 50% (IC_50_) of the PTP1B activity was determined through curves at different concentration of each compound, adjusting the data to the equation reported elsewhere [80]. The inhibition type and constant were obtained by the measurement of the initial velocities of hydrolysis varying the substrate concentration in a range of 2–30 µM, in absence or presence of fixed concentrations of each inhibitor. For compound **1** the concentrations used were 3, 6, 10 and 20 µM; in the case of compound **2** were 2, 4, 6, 8, and 10 µM; and for compound **3** were 20, 50, 70 and 90 µM. The experimental data were analyzed through the Lineweaver-Burk plot using the software Sigma Plot V12.3 (Systat Software, Inc., San Jose, CA, USA).

### 3.6. Molecular Docking

The molecules were built in Maestro 10.4 (www.schrodinger.com) and prepared in Ligprep 2.3 [81]. The crystallographic structure of the PTP1B protein was obtained from the RCSB Protein Data Bank with the code PDB ID 2F71 [64]. Hydrogen atoms were added to the structure, bond angles, and distances were corrected, and charges were assigned using Protein Preparation Wizard [82], all ions and the inhibitor present in the crystallographic structure were withdrawn. Water molecules were also withdrawn, with exception of those located in the WPD loop, since these are considered necessary to give better binding poses [83]. Energy minimization was performed with the OPLS_2005 force field with an RMSD of 0.3 Å. The molecular docking simulations in the active site of the PTP1B were performed using Glide [84,85]. The Van der Waals scale was of a factor of 0.80 and a cutting partial charge of 0.15. The files were limited to at least one pose for ligand, rejecting poses with energies smaller than 0.5 kcal/mol. Standard precision and Extra precision modes were used with flexible ligand adding penalization states of the Epik software [86] in the docking score.

### 3.7. Molecular Dynamic Simulations

The initial structures for the simulations were those with the lowest binding energy of each complex obtained by docking. The necessary topology files for each ligand (compounds **1**, **2** and **3**) were calculated and obtained using PRODRG [87]. The systems were solvated within a water box with 1.0 nm of distance from the proteins surface with the Single Point Charge (SPC) water model. Sodium and Chlorine ions were added to neutralize the systems charge until a 0.15 M concentration was reached. First, a descending steps energy minimization was done. Afterwards, a canonic assemble was performed, continuing by an isobaric-isothermal assemble, maintaining a constant temperature, volume and pressure. Finally, 10 ns simulations were performed for each complex and the free enzyme in GROMACS 5.1 software [88] using the Gromos 43a147 force field. All simulations were performed at 1 bar of pressure and 300 °K of temperature. The free binding energy was calculated based on the molecular mechanics of surface area of Poisson-Boltzmann (MM-PBSA) method [89]. 

### 3.8. Drug-Like and Toxicological Propierties

The FAF-Drugs4 server [42] and the Molsoft [45] program, available on the web were used. The Drug-like soft filter used in FAF-Drugs4 combines the physicochemical properties described in several articles and an analysis of the descriptor values of 916 oral medications of the FDA, allowing defining a filter threshold that comprises up to 90% of these drugs. The ranges of the permitted values used by the software are shown in Table 1. Regarding Molsoft, it uses the fingerprints technique with a set of 5000 commercialized drugs and 10,000 non-pharmacological compounds. After the process, it reports a score that places the molecules in a range between the parameters of the drugs and the non-drugs, which allows defining their pharmacological potential; values between −1 to 2 are accepted.

With respect to toxicological parameters, PROTOX [72] server was used, as well the Data Warrior software [73] to determine mutagenesis, tumorigenic, reproductive effects and irritability. PROTOX uses the identification of fragments over-represented in toxic compounds and similarity analyses of compounds with known LD50 values. Furthermore, based on pharmacophores, it indicates possible toxicity targets. Data Warrior uses chemical descriptors to make several molecular similarity measures and predict properties such as mutagenicity, tumorigenicity, irritant and reproductive effects. 

## 4. Conclusions

In the present work three new mixed type inhibitors for PTP1B are reported, which based on their inhibition capability and mechanism, potential selectivity against TCPTP, and predicted drug-like properties, could represent a good starting point for the development of more potent molecules that can guide the design of a new drug to treat type 2 diabetes.

## Figures and Tables

**Table 1:** The ten most potent PTP1B inhibitors.

Molecule	MW ^a^	HBD ^a^	HBA ^a^	LogP ^a^	Drug Likeness ^b^	Binding Energy (Kcal/mol)	% Inhibition (200 µM)
**1**	567.27	2	7	6.68	1.07	−4.20	100
**2**	466.96	2	6	6.00	0.86	−4.99	99
**3**	424.59	2	5	5.53	0.66	−4.47	92
**4**	356.36	1	8	2.94	-0.49	−3.70	88
**5**	496.32	0	9	5.13	0.18	−4.02	85
**6**	498.47	2	8	4.26	-0.12	−5.24	84
**7**	499.94	1	7	4.95	0.41	−3.49	80
**8**	446.51	1	5	5.80	0.05	−3.98	74
**9**	384.64	1	5	4.36	0.76	−3.99	70
**10**	356.83	1	4	4.35	0.08	−4.01	65

^a^ Server FAFDrugs [44], filter Drug-like soft was used: MW 100–600; HBD ≤ 5; HBA ≤ 12; LogP −3 to 6. ^b^ Server Molsolf [45], Drug-Likeness score was determined, values between −1 to 2 are accepted.

**Table 2:** Type of inhibition and kinetic parameters for PTP1B inhibitors.

Molecule	Ki (µM)	IC_50_ (µM)	α	V_max_ (µmol/min/mg)	Km (mM)	Inhibition Type
**1**	5.2	7.5	1.4	8.8	6.7	Mixed
**2**	4.2	8.4	2.9	16	6.8	Mixed
**3**	41.3	31.3	3.3	10	4.1	Mixed

**Table 3:** Binding free energies determined by the MMPBSA method, and hydrogen bonds of the protein-ligand complexes.

Complex	Energy (kcal/mol)					Hydrogen Bonds	
	Van der Waals Energy	Electrostatic Energy	Polar Solvation Energy	SASA Energy	ΔG Binding	Range	Average
PTP1B-**1**	−47.56	−17.97	32.34	−4.17	−37.36	0–3	3
PTP1B-**2**	−35.46	−27.01	33.57	−3.54	−32.43	0–5	4
PTP1B-**3**	−29.50	−3.63	12.33	−2.93	−23.74	0–4	2

**Table 4:** Toxicological profile of PTP1B inhibitors.

Molecule	LD50 ^a^ (mg/kg)	Toxicity Class ^a^	Toxic Frag. ^a^	Toxicity Targets ^a^	Mutagenic ^b^	Tumorigenic ^b^	Reprod. Effec. ^b^	Irritant ^b^	Drug Likeness ^c^
**1**	1600	4	None	No Binding	Low	None	High	High	1.07
**2**	1000	4	None	No Binding	None	None	None	None	0.86
**3**	1600	4	None	No Binding	Low	None	High	High	0.66

^a^ Toxicity Class was determined in Server PROTOX [72], values ranged between 1 to 6, 1 is toxic and 6 is safe. Toxicity targets were determined for: Adenosine A2A receptor, Adrenergic beta 2 receptor, Androgen receptor, Amine oxidase, Dopamine D3 receptor, Estrogen receptor 1 and 2, Glucocorticoid receptor, Histamine H1 receptor, Nuclear receptor subfamily 1 group I member 2, Opioid receptor kappa, Opioid receptor mu, cAMP-specific 3′,5′-cyclic phosphodiesterase 4D, Prostaglandin G/H synthase 1, Progesterone receptor. ^b^ Mutagenic, Tumorigenic, Reproductive effective and Irritant effects were determined using Data Warrior [73]. ^c^ Drug-Likeness score was determined with Server Molsolf [45], values between −1 to 2 are accepted.

## Supplementary Materials

Supplementary materials are available online.
