# Supplementary material for: Novel Mixed-Type Inhibitors of Protein Tyrosine Phosphatase 1B. Kinetic and Computational Studies
Source: Molecules. 2017 Dec 20;22(12):2262. doi: 10.3390/molecules22122262 (PMC6150025; doi:10.3390/molecules22122262)
Supplement: Supplementary file 1 [file molecules-22-02262-s001.pdf]

Table S1. PTP1B inhibitors with an inhibition range between 31-64% (Continue).

Structure	Name	% Inhibition (200 µM)
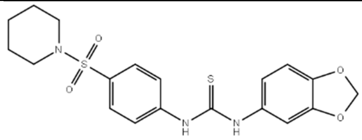	N-(1,3-benzodioxol-5-yl)-N'-[4-(piperidinosulfonyl)phenyl]thiourea	32
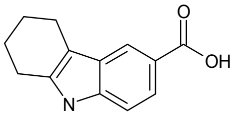	6,7,8,9-Tetrahydro-5h-carbazole-3-carboxylic acid	32
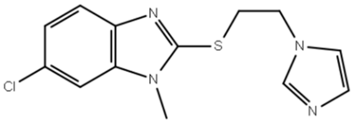	6-chloro-2-{[2-(1H-imidazol-1-yl)ethyl]sulfanyl}-1-methyl-1H-benzimidazole	32
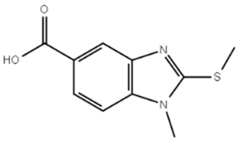	1-methyl-2-(methylsulfanyl)-1H-benzimidazole-5-carboxylic acid	33
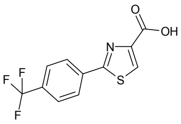	2-[4-(trifluoromethyl)phenyl]-1,3-thiazole-4-carboxylic acid	34
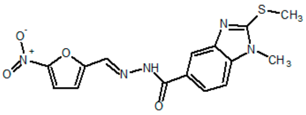	1-methyl-2-(methylsulfanyl)-N'-[(E)-(5-nitrofuran-2-yl)methylidene]-1H-benzimidazole-5-carbohydrazide	34
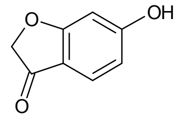	6-hydroxy-2,3-dihydrobenzo[b]furan-3-one	37
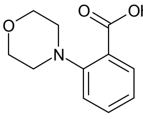	2-morpholinobenzoic acid	37
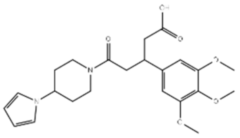	5-oxo-5-[4-(1H-pyrrol-1-yl)piperidino]-3-(3,4,5-trimethoxyphenyl)pentanoic acid	38
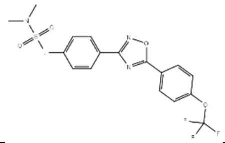	N,N-dimethyl(4-{5-[4-(trifluoromethoxy)phenyl]-1,2,4-oxadiazol-3-yl}phenyl)sulfamate	38

Table S1. Continue.

Structure	Name	% Inhibition
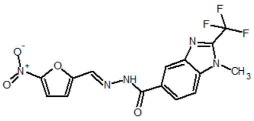	1-methyl-N'-[(E)-(5-nitrofuran-2-yl)methylidene]-2-(trifluoromethyl)-1H-benzimidazole-5-carbohydrazide	38[a]
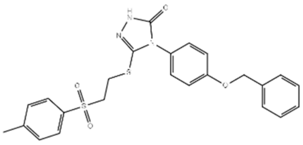	4-[4-(benzyloxy)phenyl]-5-({2-[(4-methylphenyl)sulfonyl]ethyl}sulfanyl)-2,4-dihydro-3H-1,2,4-triazol-3-one	42
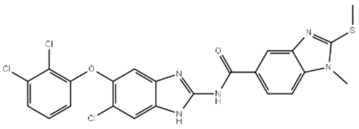	N-[6-chloro-5-(2,3-dichlorophenoxy)-1H-benzimidazol-2-yl]-1-methyl-2-(methylsulfanyl)-1H-benzimidazole-5-carboxamide	42
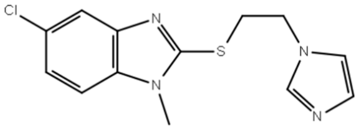	5-chloro-2-{[2-(1H-imidazol-1-yl)ethyl]sulfanyl}-1-methyl-1H-benzimidazole	42
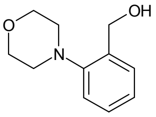	(2-morpholinophenyl)methanol	44
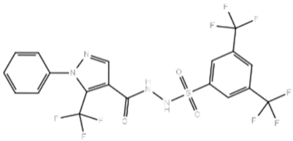	N'1-{[1-phenyl-5-(trifluoromethyl)-1H-pyrazol-4-yl]carbonyl}-3,5-di(trifluoromethyl)benzene-1-sulfonohydrazide	45
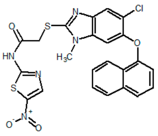	----	48[a]
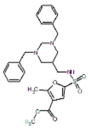	methyl 5-({[(1,3-dibenzylhexahydro-5-pyrimidinyl)methyl]amino}sulfonyl)-2-methyl-3-furoate	50
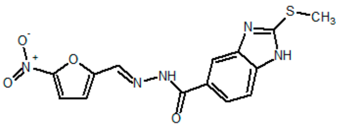	2-(methylsulfanyl)-N'-[(E)-(5-nitrofuran-2-yl)methylidene]-1H-benzimidazole-5-carbohydrazide	50[a]

Table S1. Continue.

Structure	Name	% Inhibition
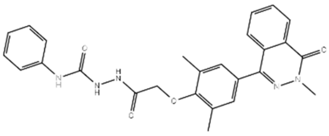	N1-phenyl-2-{2-[2,6-dimethyl-4-(3-methyl-4-oxo-3,4-dihydrophthalazin-1-yl)phenoxy]acetyl}hydrazine-1-carboxamide	52
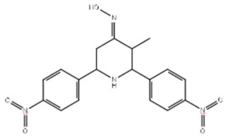	3-methyl-2,6-bis(4-nitrophenyl)tetrahydropyridin-4(1H)-one oxime	54
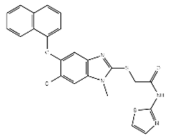	2-({6-chloro-1-methyl-5-[(naphthalen-1-yl)oxy]-1H-benzimidazol-2-yl}sulfanyl)-N-(1,3-thiazol-2-yl)acetamide	54
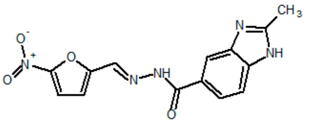	2-methyl-N'-[(E)-(5-nitrofuran-2-yl)methylidene]-1H-benzimidazole-5-carbohydrazide	54[a]
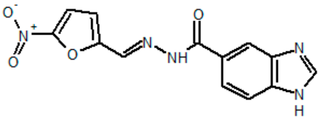	N'-[(E)-(5-nitrofuran-2-yl)methylidene]-1H-benzimidazole-5-carbohydrazide	55[a]
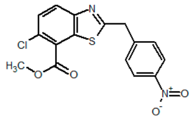	methyl 6-chloro-2-[(4-nitrophenyl)methyl]-1,3-benzothiazole-7-carboxylate	56
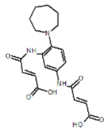	4-{2-(1-azepanyl)-5-[(4-hydroxy-4-oxo-2-butenoyl)amino]anilino}-4-oxo-2-butenoic acid	58
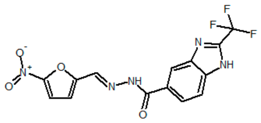	N'-[(E)-(5-nitrofuran-2-yl)methylidene]-2-(trifluoromethyl)-1H-benzimidazole-5-carbohydrazide	62[a]
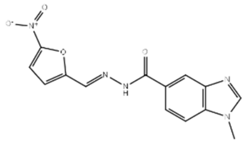	1-methyl-N'-[(E)-(5-nitrofuran-2-yl)methylidene]-1H-benzimidazole-5-carbohydrazide	62[a]
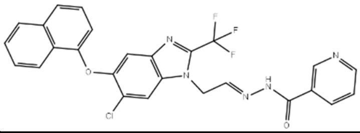	----	62[a]

Table S1. Continue.

Structure	Name	% Inhibition
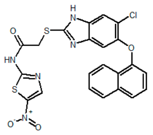	2-({6-chloro-5-[(naphthalen-1-yl)oxy]-1H-benzimidazol-2-yl}sulfanyl)-N-(5-nitro-1,3-thiazol-2-yl)acetamide	63
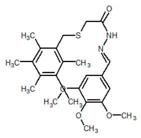	N'1-(3,4,5-trimethoxybenzylidene)-2-[(2,3,4,5,6-pentamethylbenzyl)thio]ethanohydrazide	63
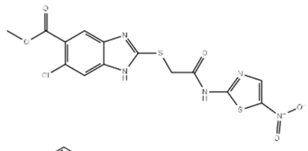	methyl 6-chloro-2-({2-[(5-nitro-1,3-thiazol-2-yl)amino]-2-oxoethyl}sulfanyl)-1H-benzimidazole-5-carboxylate	63
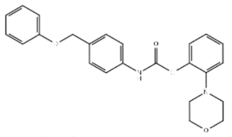	2-morpholinophenyl N-[4-(phenoxymethyl)phenyl]carbamate	63
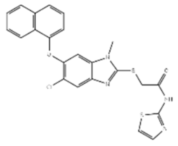	2-({5-chloro-1-methyl-6-[(naphthalen-1-yl)oxy]-1H-benzimidazol-2-yl}sulfanyl)-N-(1,3-thiazol-2-yl)acetamide	63[a]
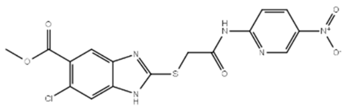	methyl 6-chloro-2-({2-[(5-nitropyridin-2-yl)amino]-2-oxoethyl}sulfanyl)-1H-benzimidazole-5-carboxylate	63
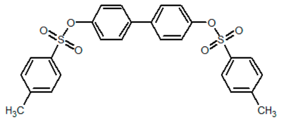	4'-{[(4-methylphenyl)sulfonyl]oxy}[1,1'-biphenyl]-4-yl 4-methylbenzenesulfonate	64
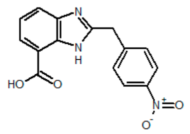	2-[(4-nitrophenyl)methyl]-1H-benzimidazole-7-carboxylic acid	64
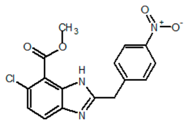	methyl 6-chloro-2-[(4-nitrophenyl)methyl]-1H-benzimidazole-7-carboxylate	64
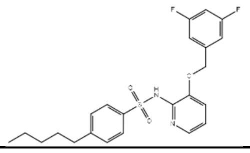	N-{3-[(3,5-difluorobenzyl)oxy]pyridin-2-yl}-4-pentylbenzenesulfonamide	64

Table S1. Continue.

Structure	Name	% Inhibition
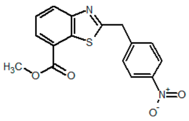	methyl 2-[(4-nitrophenyl)methyl]-1,3-benzothiazole-7-carboxylate	64
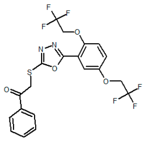	2-({5-[2,5-di(2,2,2-trifluoroethoxy)phenyl]-1,3,4-oxadiazol-2-yl}thio)-1-phenylethan-1-one	64
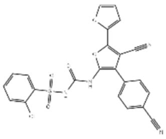	2-[({[(2-chlorophenyl)sulphonyl]amino}carbonyl)amino]-4-cyano-3-(4-cyanophenyl)-5-(2-furyl)furan	64

[1] These compounds were tested at 100 μM due to solubility problems.

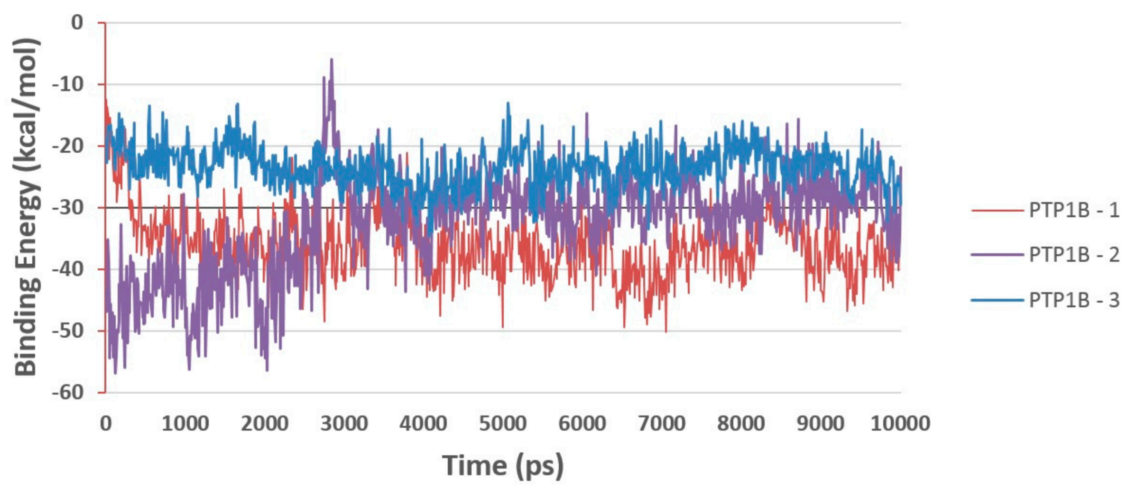

Figure S1. Total binding energy variation of the PTP1B-inhibitor complexes. The image shows that the energy in the three cases remains constant along the entire simulation, indicating structural stability of the complexes.

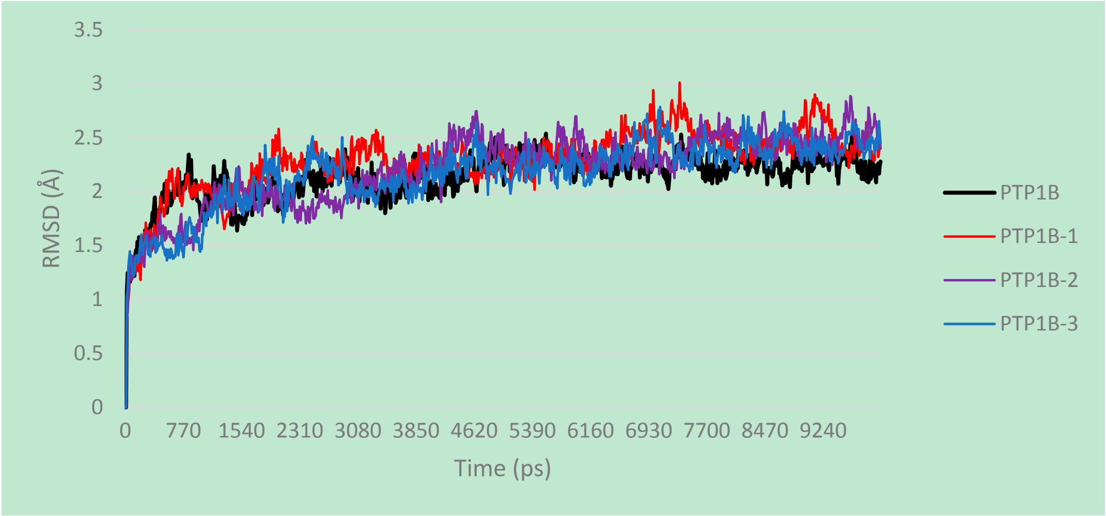

Figure S2. Ligand Positional RMSD of the PTP1B-inhibitor complex during 10 ns of simulation. Image shows that after 4 ns all the complexes reached the stability.

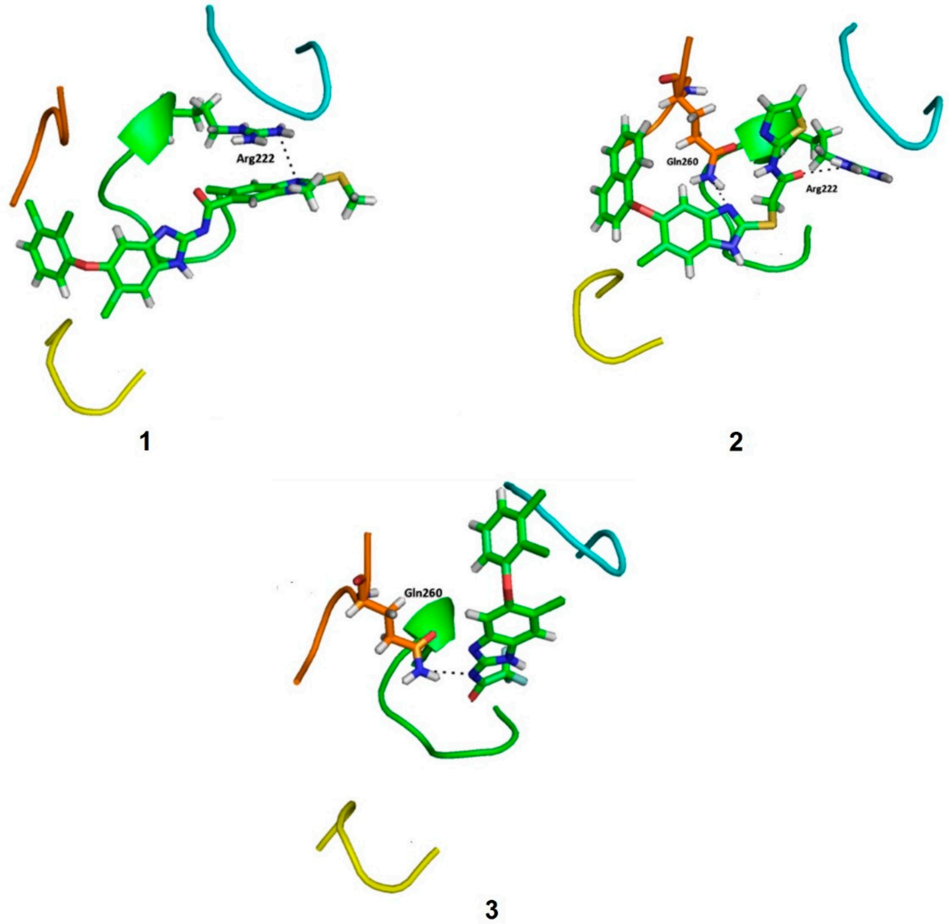

Figure S3. Binding mode of compounds **1**, **2** and **3** in TCPTP. Loops are highlighted as follows: P loop (green), WPD loop (cyan), Q262 loop (orange), and pTyr46 loop (yellow).
